# Encapsulation and release of non-fluorescent crystal violet confined in bile-salt aggregates†

**DOI:** 10.1039/d0ra06599d

**Published:** 2021-03-15

**Authors:** Prachi Sharma, Neeraj Sohal, Banibrata Maity

**Affiliations:** School of Chemistry and Biochemistry, Thapar Institute of Engineering and Technology Patiala 147004 India; School of Chemistry and Biochemistry, Affiliate Faculty-TIET-Virginia Tech Center of Excellence in Emerging Materials, Thapar Institute of Engineering and Technology Patiala-147004 India banibrata.maity@thapar.edu baniiitchem@gmail.com

## Abstract

In this work, the entrapment of non-fluorescent dye Crystal Violet (CV) in presence of bio-mimetic confined bile-salt aggregates has been studied. The photophysical characteristic properties of CV have been carried out by changing different kinds of hydrophilic head groups and hydrophobic skeletons of bile-salt aggregates (NaC, NaDC, NaTC and NaTGC). The main aim of this work is to modulate the solubility behaviour, fluorescence properties and elucidation of different kinds of non-covalent interaction of CV confined in bile-salt aggregates. To interpret the result, steady state absorption and fluorescence emission techniques have been employed. In aqueous buffer, the CV molecule is non-fluorescent in nature. The value of fluorescence quantum yield (*Φ*) is ∼10^−4^. It has been observed that CV confined in bile-salt aggregates becomes highly fluorescent in nature. The enhancement of ‘*Φ*’ value of CV in bile-salt aggregates is ∼1000 fold compared to that of aqueous buffer medium. It has also been observed that in the presence of different bile-salt aggregates, CV exhibits remarkable enhancement of absorption and fluorescence emission spectral behaviour. The ground state and the excited state binding constant values of CV in the presence of different bile-salt aggregates have been determined. Moreover, the release of the dye molecule from the confined bile-salt aggregates to the aqueous medium has been executed. It has been found that addition of a very minute concentration of KCl salt (100 nm) to the bile-salt aggregates leads to extreme modification of their photophysical properties of CV. The absorption, fluorescence intensity, fluorescence quantum yield, ground state and excited state binding constant values, partition coefficient and aggregation number of CV molecules entrapped in bile-salt aggregates significantly reduces by addition of KCl. This result clearly confirms that CV releases from the confined system to the aqueous medium.

## Abstract 2

In this work, the entrapment of non-fluorescent dye Crystal Violet (CV) in presence of bio-mimetic confined bile-salt aggregates has been studied.

## Graphical abstract

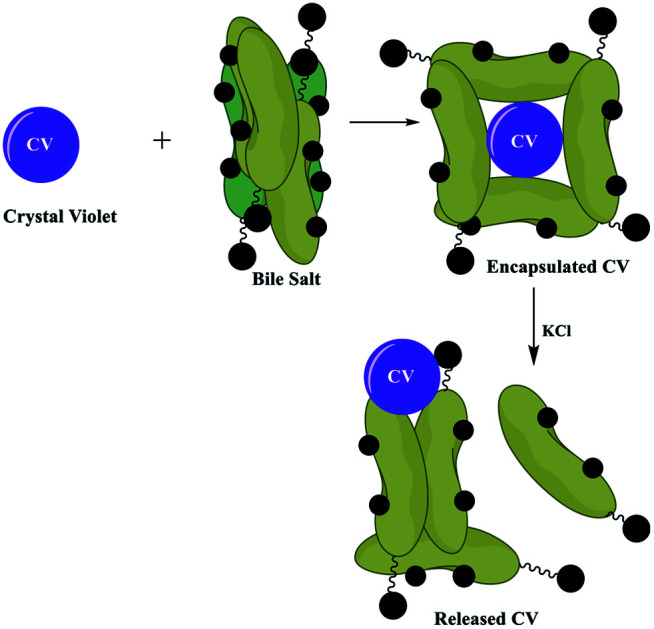

## 1. Introduction

Bile salts are naturally occurring non-conventional bio-surfactants, synthesized with the help of a cytochrome named P450 through well-moderated oxidation of cholesterol in liver in the form of greenish yellow secretion commonly known as bile. This bile is composed of different constituents, which are deoxycholic, cholic, chenodeoxycholic, and lithocholic acids. These acids interact with taurine or glycine, forming various complex bile salt derivatives. Bile salts are also called amphiphilic surfactants. Due to the presence of small polar head groups and flexible non-polar tail groups, bile-salts exhibited a rigid structure. Bile-salts have a convex side of their steroid moiety, which is hydrophobic in nature and a concave side, which is made up of hydrophilic polar groups (generally hydroxyl). This facial amphiphilicity imparts a unique property in bile-salts which leads to selective binding with different molecules. This properties of bile-salts can be exploited in the medicinal field.^1–7^

**Scheme 1:**
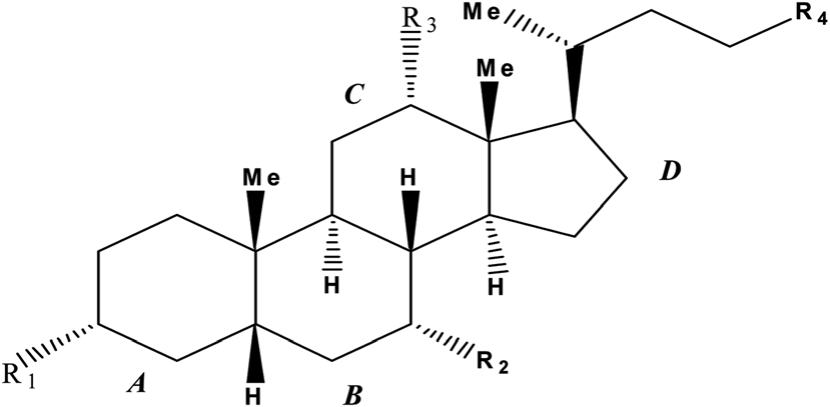
Schematic representation of carbon backbone of bile-salts.

Bile-salts (Scheme 1) are easily recognized by its four-membered hydrocarbon core known as “cyclopentanoperhydrophenanthrene”. All the natural as well as synthetic steroids are derivatives of this core. Ring ‘A’ is the cyclohexane ring which is fused to another six-membered ring called ring ‘B’, followed by ring ‘C’ and a cyclopentane system called ring ‘D’. On the basis of different number, position and orientation of hydroxyl group at C-3, C-7 and C-12 position of the steroid ring human bile-salts are categorized as various names (tabulated in Table 1).

**Table 1:** Nomenclature of different bile-salts

Bile-salts	R_1_	R_2_	R_3_	R_4_
Sodium cholate (NaC)	α-OH	α-OH	α-OH	–COOH
Sodium taurocholate (NaTC)	α-OH	α-OH	α-OH	–CONHCH_2_CH_2_SO_3_H
Sodium deoxycholate (NaDC)	α-OH	-H	α-OH	–COOH
Sodium tauroglycocholate (NaTGC)	α-OH	α-OH	α-OH	–CONHCH_2_CONHCH_2_CH_2_SO_3_H

The biological importance of bile-salts is their capability to solubilize fats, monoglycerides, and emulsification of cholesterol, as well as fat-soluble vitamins in a human digestive system.^1–3^ Bile salts play an important role in assisting the digestion of lipids and fats inside human system. Apart from these physiological applications, bile salt aggregates play a pivotal role in pharmaceutical and biochemical applications as vehicles for carrying many sparingly water soluble drug molecules. With the increase in demand for eco-friendly biodegradable micro-encapsulators bile salts have been used to dissolve hydrophobic probes making them an excellent host systems.^8,9^ While selecting an ideal drug carrier one has to consider several biopharmaceutical factors like drug stability, solubility in the gastrointestinal fluids, metabolic stability and sufficient intestinal permeability for absorption of drug molecule. Unfortunately, more than 50% of drug molecules have low aqueous solubility.^4^ The enterohepatic organotropism of bile-salts convert them into intriguing drug delivery vehicles for targeting these selective drugs and enhancing bioavailability of the drug molecule by improvising their intestinal absorption and metabolic stability. Therefore such compounds are well solubilised in bile salts and thus can be easily transported to different parts of body resulting in increase of their bio-availability.^4^ Due to their biocompatible and biodegradable nature bile-salts have gained a lot of attention for various drugs, cosmetic materials, vitamins *etc.* making them very cheap, non-toxic and efficacious drug carrier vehicle for medical applications.^5,6^ Bile salts have tendency to form micelles and aggregation behaviour beyond their critical micelle concentration (CMC). Bile salt aggregates are typically used as supramolecular host molecules. As a result, several biological active fluorophores (guest) are entrapped in their caged structure and forms encapsulated supramolecular host–guest complex. Numerous photophysical studies of encapsulated molecules confined in bile-salt aggregates are reported in literature.^8–18^

**Scheme 2:**
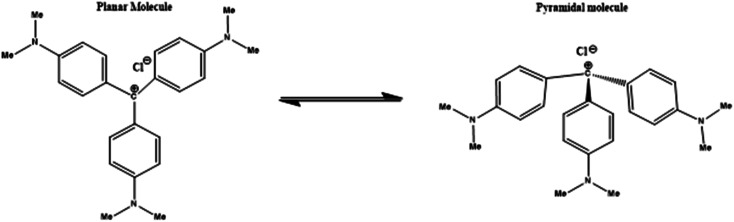
Schematic representation of two isomeric form of crystal violet.

Triphenylmethane dyes are generally known as aniline dyes. They are one of the most important classes of commercial dyes, which have a wide variety of potential applications in photographic industry, dyeing, and textile industry, sensitizers for photoconductivity in environmental science, medicinal and biological chemistry.^19–22^ Crystal violet exhibits potent clinically useful properties and exhibits various pharmacological activities.^23–26^ CV is one of the most important dye molecule which is used for the histological staining, classifying bacteria (Gram's method), topical treatment of skin infections and wounds like burns, ringworm and eye infections like pink eye, due to its antiseptic, anti-fungal, anti-bacterial properties. From almost 100 years, it has been used for medicinal treatment for anti-angiogenic, anti-trypanosomal, anti-helminthic and anti-tumour activities. Recently it has also been used as a blood additive during the transfusion to inactivate *Trypanosoma cruzi* (causative agent), for the prevention of the transmission of Chagas' disease.^15^ CV can also be used for the treatment of *Ichthyophthirius multifiliis*, which leads to ‘white spot disease’ in fish. Crystal violet (also known as Gentian violet or methyl violet 10B) is chemically assigned as tris(4-(dimethylamino)phenyl)methylium chloride. It is an inexpensive cationic dye (Scheme 2).

X-Ray studies indicate that the structure of CV molecule is propeller-shaped with its aromatic rings rotated by 32° from the central horizontal plane.^21^ In aqueous solution, CV molecule is surrounded by water molecules and chloride counter ions, which interact with the positive charge present on central C and N of CV molecule and results in changes in symmetry.^27^ This dark purple coloured dye (due to its conjugative structure) shows striking optical behaviour as it exhibits intense visible absorption bands of molar extinction coefficient (*ε*_max_) is 10^5^ cm^2^ M^−1^ upon excitation. In aqueous medium, CV molecule exhibits weak fluorescence due to very fast rotation of its aromatic rings. The absorption spectrum of CV displays one shoulder band along with the absorption maxima. This characteristic feature of the absorption spectra of CV can be explained on the basis of the existence of two ground state isomers. The two type of ground state isomers (Scheme 2) are planar form and the distorted or the pyramidal form.^22^

Lum *et al.* reported Surface-Enhanced Raman Spectroscopy (SERS) substrates of encapsulated crystal violet inside the aqueous liposome medium. They also confirmed that in presence of liposome, high SERS enhancement of the studied biological probe molecule are encapsulated.^28^ Revuelta *et al.* studied the encapsulation of crystal Violet molecule in a biopolymeric matrix composed of pectin and Arabic gum.^29^ Very limited studies on interaction of CV in confined environments have reported in literature.^22,28–30^ Therefore, the main aim and motivation of this work is to endeavour the interaction of CV in confinement of different kinds of bile-salt aggregates. Since, CV is non-fluorescent in aqueous medium; therefore another aim of this study is to improve the fluorescence property of CV due to supramolecular interactions in confinement of bile salt aggregates. Therefore, to get more insight and comprehend the interactions of encapsulated complex, the photophysics of CV molecule have been carried out by modulating several kinds of hydrophilic head groups and hydrophobic skeletons of bile-salt aggregates (*e.g.* NaC, NaDC, NaTC and NaGDC) and to rationalize the location of CV molecule in confined environment. Another major aim of this work is to release the CV molecule from encapsulated bile-salt aggregates to the aqueous medium by addition of foreign substance (non-toxic and green method). This will be possible if the studied CV molecule will exhibits strong fluorescence to non-fluorescence property or in other words, fluorescence turn-on-off property. The detection analysis of the bio-mimetic confined bile-salt aggregates on the studied biologically active CV molecule and its release phenomenon is very much important in biological model systems. Addition of KCl salt perturbs the micellization process of bile-salt aggregates. As a result, CV molecule releases from the confined environments to aqueous medium.

## 2. Experimental section

Crystal Violet (CV) was purchased from Loba Chemie, India and used as received. The purity of the compounds was more than 97%. Fresh solution of CV was prepared every time to avoid any problem of degradation and aggregation. The concentration of CV solution was maintained at 1 × 10^−5^ (M). Bile-salts such as sodium cholate (NaC), sodium deoxycholate (NaDC), sodium taurocholate (NaTC) and sodium tauroglycocholate (NATGC) were obtained from Loba Chemie. Potassium chloride (KCl) was obtained from Sigma-Aldrich, India. All the bile-salts used of high purity grade (≥97%).

Absorbance measurements were performed by Specord 205 Analytik Jena spectrophotometer, India using 1 cm path length quartz cuvette. The spectra were recorded for 400–800 nm wavelength range. The fluorescence emission spectra of the experimental solution were measured by PerkinElmer LS 55 fluorescence spectrometer, USA using quartz cuvette of a 1 cm path length. Fluorescence spectra were recorded at two different excitation wavelengths (*λ*_exi_ = 550 nm and 590 nm) two different excitation wavelengths were selected since the studied dye molecule displayed shoulder band (550 nm) followed by absorption maxima (590 nm). The emission slit widths were fixed at 15 nm and 15 nm respectively. The scan time was fixed at 250 nm per minute. Fourier transform infrared (FT-IR) spectral data were recorded by PerkinElmer Spectrum 400 instrument, USA in attenuated total reflection (ATR) mode with diamond crystal having resolution of 2 cm^−1^. FE-SEM image was recorded using Hitachi S4800 instrument, Japan with an acceleration voltage of 10.0 kV. All the experiments were performed at physiological pH value of 7.4 by using 0.01 M phosphate buffer solution.

Fluorescence quantum yield values are determined from the fluorescence emission intensity (integrated area) and the absorbance value at the particular wavelength of excitation. The fluorescence quantum yield can be mathematically expressed as:^31^
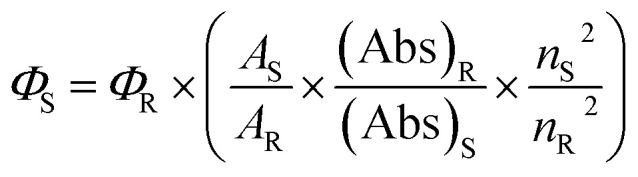
where, ‘*Φ*_S_’ and ‘*Φ*_R_’ represents the fluorescence quantum yield of sample (CV) and reference (Rhodamine B), ‘Abs’ denotes absorbance, ‘*A*’ represents the area under the fluorescence emission, ‘*n*’ is the refractive index of the solvent used. The subscripts ‘S’ and ‘R’ denotes the corresponding parameters for the CV (sample) and Rhodamine B (reference) respectively. The fluorescence quantum yields of CV in different bile-salt systems were determined by using ‘Rhodamine B' as reference solution in aqueous medium (*Φ*_R_ = 0.31).^32^

## 3. Results and discussion

In phosphate buffer solution, CV showed shoulder band ∼550 nm along with the absorption maxima at 590 nm. The origin of the shoulder band of CV has remained a topic of argument from past many years. Lueck *et al.*^27^ proposed that the appearance of the shoulder band was due to formation of dimeric structure in which the dimethylamino groups overlap in a head-to-tail fashion resulting in increased hydrophobic interactions which are the driving forces for dimer formation. Garcia-Rio *et al.*^33^ explained the presence of CV shoulder band is due to the existence of two ground state isomers in the aqueous medium, one is the pyramidal form (*C*_3_ symmetry) or distorted form, which is caused by rotation of phenyl rings and another is propeller structure (*D*_3_ symmetry).

**Fig. 1:**
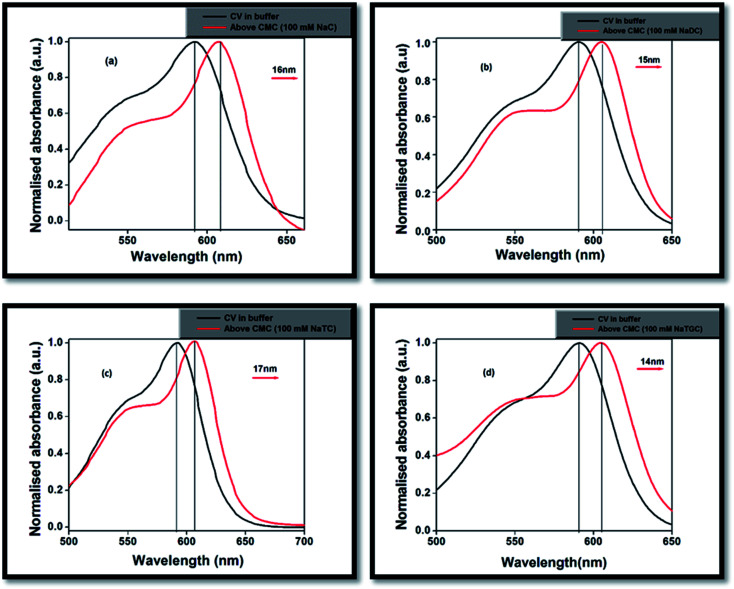
Normalised absorption of spectra of CV in (a) NaC, (b) NaDC, (c) NaTC and (d) NaTGC bile-salts.

**Fig. 2:**
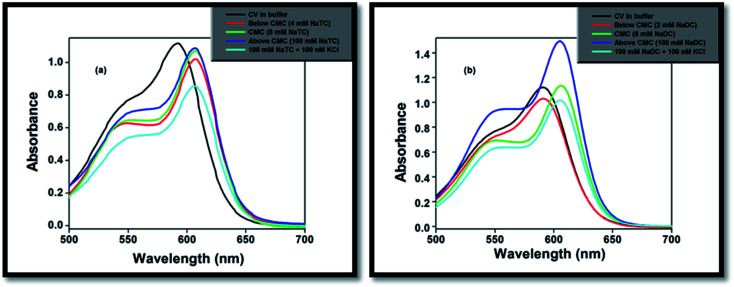
Absorption studies of CV on gradual addition of (a) NaTC and (b) NaDC in phosphate buffer medium.

On gradual addition of the respective bile-salts, CV molecule undergoes significant bathochromic shift (Fig. 1) as well as enhancement of the absorbance values (Fig. 2). The enhancement of the absorbance values of CV in the presence of bile-salt aggregates is a clear manifestation that the micro-environment around CV molecule has been modified due to formation of bile-aggregates.^34,35^ The specific interactions or changes in the micro-environment around the dye molecule might occur, which can cause redistribution of electron densities in the chromophore leading to the spectral changes. This result clearly suggested that electrostatic interactions takes places between the cationic dye molecule with the hydrophilic anionic parts of the respective bile-salts. The enhancement of the absorbance value signified that the extent of solubility of CV molecule confined in bile-salt aggregates significantly enhances due to hydrophobic–hydrophobic interaction. Due to the presence of strong hydrophobic aromatic group of CV, it has tendency to dissolve in hydrophobic core of the bile-salts. Therefore, both the electrostatic and hydrophobic interactions occur between CV and bile-salt aggregates. This analysis may also give a preliminary idea that the dye molecule encapsulate to the confined bile-salt aggregates from the aqueous solution. From literature reports, we have also found that various hydrophobic guest molecules are encapsulated to the hydrophobic interiors of the bile-salt aggregates, which caused difference of the absorbance values.^11,16–18,34.35^

The absorbance value of the CV–bile aggregates astonishingly decreases on progressive addition of lower concentration (100 nM) KCl salt (Fig. 2). The decrease of the absorbance value may be due to the reason that the solubility of the dye molecule becomes comparatively less than the solubility of the dye molecule entrapped in bile-salt aggregates. Since, the dye molecule is hydrophobic in nature. Therefore, in bile-salt aggregates hydrophobic–hydrophobic interaction occurs which leads to encapsulate CV molecule. In presence of KCl, the dye molecule may perturbs CV–bile complex and release from the confined hydrophobic core of the bile-salt aggregates to the hydrophilic regions and/or to the aqueous medium. As a result, comparatively less interaction of the dye molecule occurs upon addition of KCl salt. It is noteworthy to mention that at gradual addition of KCl salt to the CV–bile aggregates, beyond 100 nM (higher concentration KCl); there is no change on the absorption spectra of CV. Therefore, from this study it may be concluded that lower concentration of salt senses the release of the drug molecule from the confined environments.

In phosphate buffer, the studied drug molecule (CV) displayed unstructured fluorescence emission maxima and the fluorescence quantum yield (*Φ*) was very low (∼10^−4^) at both the excitation wavelengths (*λ*_exi_ = 550 nm and 590 nm). Therefore, the dye molecule present in buffer solution becomes non-fluorescent in nature. Since, the studied molecule showed shoulder band (550 nm) along with the absorption maxima (590 nm) in phosphate buffer as well as in aqueous medium. Therefore, CV molecule was excited at both the selected wavelengths to comprehend the excited state dynamics and the nature of interaction of the fluorophore entrapped in bile-salt aggregates.

**Fig. 3:**
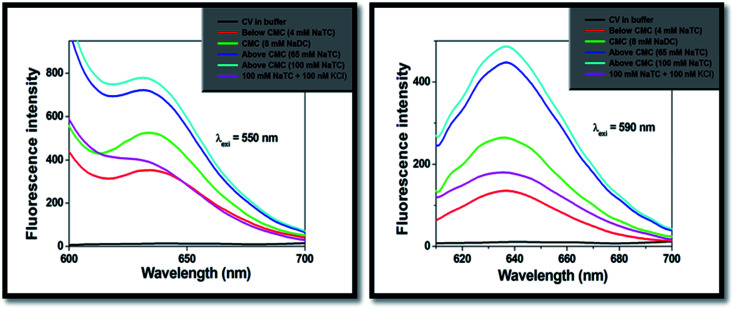
Fluorescence emission spectra studies of CV with increasing concentration of NaTC at different excitation wavelengths (*λ*_exi_ = 550 nm and 590 nm).

On progressive incorporation of the respective bile-salts to the buffer solution, the fluorescence intensity of the studied molecule (CV) at both the excitation wavelengths significantly enhanced. This characteristic modification of the emission spectra clearly demonstrated that the microenvironment of the studied molecule inside the bile-salt medium gets modulated compared to that buffer medium. Fig. 3 depicts the fluorescence intensity of CV molecule with varied concentration of NaTC bile-salts (below CMC, at CMC and highest CMC values).

The fluorescence quantum yield values (*Φ*) of CV in different bile-salt aggregates significantly enhanced (∼1000 folds) (Table 2). This result clearly suggests that CV molecule becomes strong fluorescence in nature confined in encapsulated bile-salt aggregates. From the results, it may be demonstrated that gradual addition of the respective bile-salts have tendency to agglomerate the dye molecule through hydrophobic interaction.

**Table 2:** Fluorescence quantum yield values (*Φ*) of CV in different systems

System	*Φ*_550 nm_	*Φ*_590nm_
CV (10^−5^ M) in buffer	6.79 × 10^−4^	6.54 × 10^−4^
CV (10^−5^ M) + KCl (100 nM)	1.98 × 10^−3^	1.98 × 10^−3^
CV (10^−5^ M) + NaC (100 mM)	0.12	1.6 × 10^−2^
CV + NaC (100 mM) + KCl (100 nM)	0.12	2.5 × 10^−2^
CV + KCl (100 nM) + NaC (100 mM)	9 × 10^−2^	2.6 × 10^−2^
CV (10^−5^ M) + NaDC (100 mM)	0.27	0.18
CV + NaDC (100 mM) + KCl (100 nM)	9.8 × 10^−3^	1.8 × 10^−3^
CV + KCl (100 nM) + NaDC (100 mM)	1.96 × 10^−3^	1.52 × 10^−3^
CV (10^−5^ M) + NaTC (100 mM)	0.52	0.19
CV + NaTC (100 mM) + KCl (100 nM)	4.54 × 10^−3^	1.72 × 10^−3^
CV + KCl (100 nM) + NaTC (100 mM)	9.6 × 10^−3^	2.4 × 10^−3^
CV (10^−5^ M) + NaTGC (100 mM)	0.10	1.54 × 10^−2^
CV + NaTGC (100 mM) + KCl (100 nM)	2.4 × 10^−3^	1.3 × 10^−3^
CV + KCl (100 nM) + NaTGC (100 mM)	3.84 × 10^−3^	1.71 × 10^−3^

The addition of lower concentration of KCl salt (100 nM) to the encapsulated bile-salts causes remarkable decrease of fluorescence intensity (Fig. 3) and fluorescence quantum yields (Table 2). From literature,^31^ it has been found that incorporation of salts to the bile-aggregates results more aggregation of the bile-salts, leading to enhancement of the fluorescence intensity and fluorescence quantum yield values. They also explained that addition of salts also responsible for the conformational and structural change of the bile-aggregates.^36^ But in our case, opposite result was found. Increasing the concentration of KCl salt beyond 100 nM, there is not found any change of the fluorescence intensity and fluorescence quantum yield values. This exciting result may be due to the reason that the studied drug molecule may disrupts CV–bile complex and release from the confined hydrophobic core of the bile-salt aggregates to the hydrophilic regions and/or to the aqueous medium. Similar kind of phenomenon was also obtained from the absorption study. Here, it is important to note that if the drug molecule (CV) releases from the confined bile-aggregates after the addition of small concentration of KCl salt, then the binding constant of the drug–bile aggregates should be significantly lowered.^37^

In order to get more insight the stability of the studied drug molecule (CV) in bile-salt aggregates, the binding constant values of CV molecule was evaluated by non-linear 1 : 1 regression analysis method:^38^
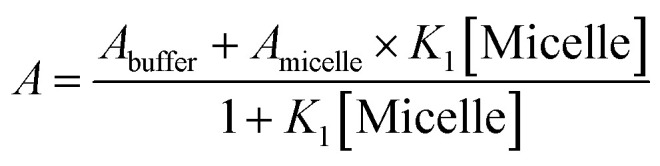
where, ‘*A*_buffer_’ and ‘*A*_micelle_’ are the absorption intensities of CV in buffer and respective highest micellar concentration of bile-salts. ‘*K*_1_’ is ground state 1 : 1 binding constant value of CV–bile aggregates.

The ground state binding constant values were calculated from the absorbance data of CV with different concentration of the respective bile-salts and are tabulated in Table 3.

**Table 3:** Binding constant values of CV in different bile-salt aggregates from absorption study

Bile-salt [100 mM]	Binding constant (M^−1^) of CV–bile-salt (absence of KCl)	Binding constant (M^−1^) of CV–KCl–bile-salt (presence of KCl)
NaC	24 ± (6)	19 ± (4)
NaDC	50 ± (10)	32 ± (7)
NaTC	80 ± (21)	42 ± (10)
NaTGC	26 ± (7)	14 ± (3)

**Fig. 4:**
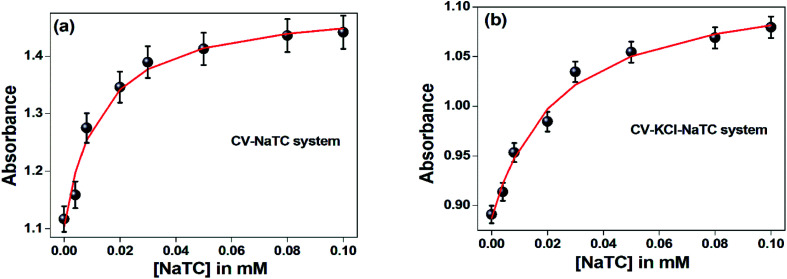
Ground state binding constant plot of (a) CV–NaTC and (b) CV–KCl–NaTC.

Similarly, in presence of KCl (100 nM), the binding constant values of CV with varying concentration of CV were also evaluated and tabulated in Table 3. From the table, it has been found that presence of KCl salt results decrease of the binding interaction between CV–bile aggregates. Fig. 4 represents the binding constant plot of CV–NaTC and CV–KCl–NaTC.

The excited state binding constant values of CV–bile aggregates in absence of KCl and in presence of KCl were also obtained from the fluorescence intensity data with varying the concentration of bile-salts using the following equation:^38^
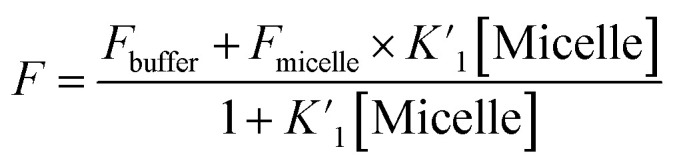
where, ‘*F*_buffer_’ and ‘*F*_micelle_’ are the fluorescence intensities of CV in buffer and respective highest micellar concentration of respective bile-salts. 
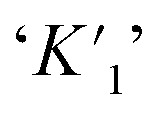
 is the excited state 1 : 1 binding constant value of CV–bile aggregates.

From Table 4, it was also clear that at two different excitation wavelengths (*λ*_exi_ = 550 nm and 590 nm), the presence of KCl salt suppress the binding interaction between CV–bile aggregates in the excited state. From the analysis of both the ground and the excited state binding studies, it can be clearly demonstrated that addition of salt drives out the drug molecule from the confined hydrophobic region of bile-aggregates to outside. As a result, binding constant values significantly dropped both in ground state and the excited state. The high binding constant or association constant of NaTC is also supported by previously reported work by Bohne *et al.*^39^ where association rate constant of different bile salt were observed in order of NaTC > NaDC > NaC.

**Table 4:** Binding constant values of CV in different bile-salt aggregates from fluorescence study at two different excitation wavelengths (*λ*_exi_ = 550 nm and 590 nm)

Bile-salt [100 mM]	*λ*_exi_ = 550 nm		*λ*_exi_ = 590 nm	
	Binding constant (M^−1^) of CV–bile salts (absence of KCl)	Binding constant (M^−1^) of CV–KCl–bile salts (presence of KCl)	Binding constant (M^−1^) of CV–bile salts (absence of KCl)	Binding constant (M^−1^) of CV–KCl–bile salts (presence of KCl)
NaC	110 ± (16)	75 ± (10)	60 ± (11)	35 ± (7)
NaDC	189 ± (25)	114 ± (17)	93 ± (14)	53 ± (11)
NaTC	206 ± (31)	69 ± (7)	103 ± (15)	54 ± (2)
NaTGC	92 ± (6)	44 ± (7)	78 ± (5)	47 ± (5)

**Fig. 5:**
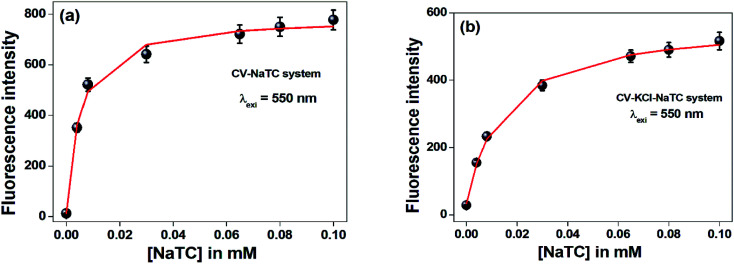
Excited state binding constant plot of (a) CV–NaTC and (b) CV–KCl–NaTGC at *λ*_exi_ = 550 nm.

It was also noticed that the extent of binding interaction at the excitation of shoulder band (*λ*_exi_ = 550 nm) is greater compared to excitation of absorption maxima band (*λ*_exi_ = 590 nm). Fig. 5 and Fig. S1† depicts the binding constant plot of one representative CV–bile-salt aggregates in absence (CV–NaTC) and in presence of salt (CV–KCl–NaTC) respectively.

To elucidate the location of the studied drug molecule (CV) at highest micellar concentration of the respective bile-salt aggregates (100 mM), the ground state and excited state partition-coefficient values were evaluated. The partition coefficient (*K*_P_) of the molecule between two different phases (aqueous and confined) is mathematically expressed as following:^16,40^
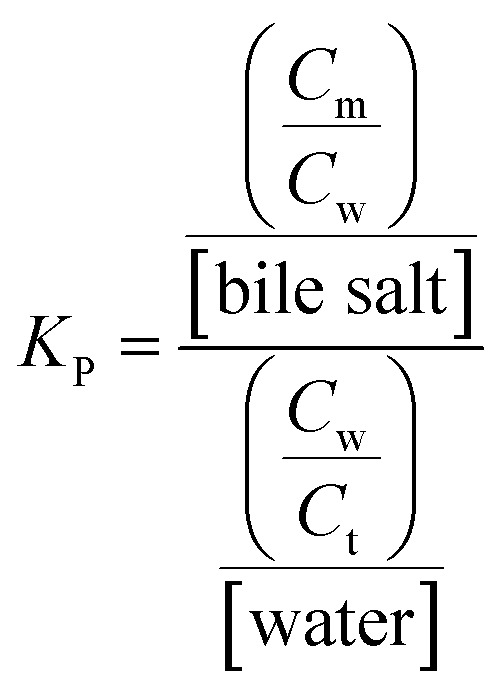
where, ‘*C*_t_’, ‘*C*_m_’ and ‘*C*_w_’ represents total concentration of dye molecule, concentration of dye bile-salt aggregates and buffer medium respectively. Experimentally, the partition coefficient^41^ can be determined from absorbance (ground state partition coefficient) as well as fluorescence intensity (excited state partition coefficient) data of CV in buffer with varying concentration of bile-salts using the following equation:^16^
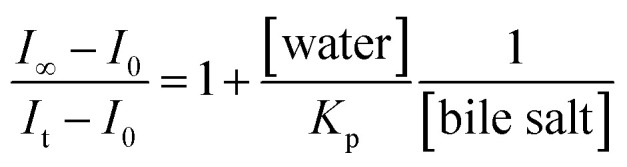
where, ‘*I*_0_’, ‘*I*_t_’ and ‘*I*_∞_’ represents the absorption and/or emission intensities of the dye molecule in aqueous buffer medium, at different concentrations (above their CMC values) of respective bile-salts and at highest micellar concentrations. ‘*K*_P_’ is the partition coefficient value. The partition coefficient values were tabulated in Table 5.

**Table 5:** Partition coefficient values of CV in different bile-salt aggregates

Bile-salt [100 mM]	Ground state		Excited state (*λ*_exi_ = 550 nm)	
	Partition coefficient (*K*_P_) of CV–bile in M^−1^ (absence of KCl)	Partition coefficient (*K*_P_) CV–KCl–bile in M^−1^ (presence of KCl)	Partition coefficient (*K*_P_) of CV–bile in M^−1^ (absence of KCl)	Partition coefficient (*K*_P_) CV–KCl–bile in M^−1^ (presence of KCl)
NaC	1748	76	8546	4751
NaDC	2112	489	14 317	5668
NaTC	1903	1791	10 540	3703
NaTGC	1804	1385	5903	2708

It was observed that magnitude of partition coefficient is very high (in order of 10^3^). This significantly higher values of partition coefficient clearly suggest that the drug molecule resides at the confined environment rather than the aqueous medium.

The partition coefficients values are in the order of NaDC > NaTC > NaTGC > NaC. Thus NaTC and NaDC have high binding as well as partition coefficient, which is also supported by various literature^42^ as NaDC due to its high hydrophobicity index forms larger aggregates and stronger complex with different probes as compared to other NaC. The hydrophobicity index of NATC, NaDC and NaC are 0, 0.72 and 0.13 respectively.^43^ Since CV exists in two isomeric form, it might be possible that the two forms binds in different fashion with amphiphilic bile-salts, where electrostatic interaction due to cationic form of CV is responsible for higher binding and partitioning for NaTC, while the hydrophobic interactions due to the presence of aromatic hydrophobic moieties of CV molecule are responsible for higher binding efficiency as well as partition coefficient for NaDC.

From the Table 5, it has been noticed that addition of KCl results significant decrease of the respective partition coefficient values both in ground as well as excited state. This clearly demonstrated that addition of KCl salt to the CV–bile aggregates the studied drug molecule comes from the confined hydrophobic environments to the aqueous medium.

**Fig. 6:**
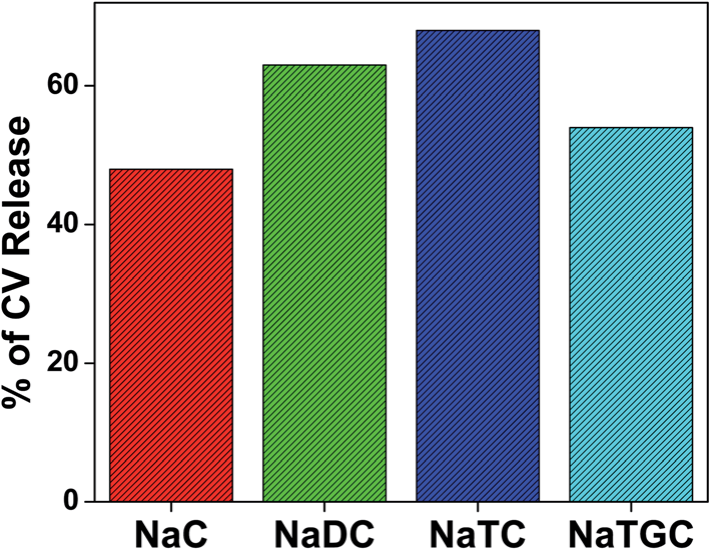
Release profile of CV molecule from different bile salts with addition of KCl salt.

Addition of KCl to the respective bile salts drives out the studied drug molecule (CV) from confined environment to the surface. Therefore, the release of drug molecule from the confined environment of bile-salts has been carried out using the fluorescence intensity data. The percentage of the release of CV molecule in different bile salt aggregates are tabulated in Table 6 and Fig. 6.

**Table 6:** Percentage (%) of release of CV molecule from different bile-salts

Bile-salts	Percentage (%) of release
NaC	48
NaDC	63
NaTC	68
NaTGC	54

From the above Table 6, it has been found that the release order is NaTC > NaDC > NaTGC > NaC. From the binding constant data (Table 3), we have also found the same trend. Therefore after analysing it has been found that more strongly bound bile-salt have propensity to release the drug molecule.

It is noteworthy to mention that we have kept the concentration of CV molecule and different bile salts as 10^−5^ M and 100 mM respectively. 0.01% CV molecule was loading in capsules. The encapsulation efficiency was 98%. From FESEM image, the size of the capsule is 50 nm. Fig. S2† represents the FESEM image of CV–NaTC bile salts. Moreover, from FTIR study, significant differences of the peak position have been observed in CV–NaTC bile salts (Fig. S3†).

**Fig. 7:**
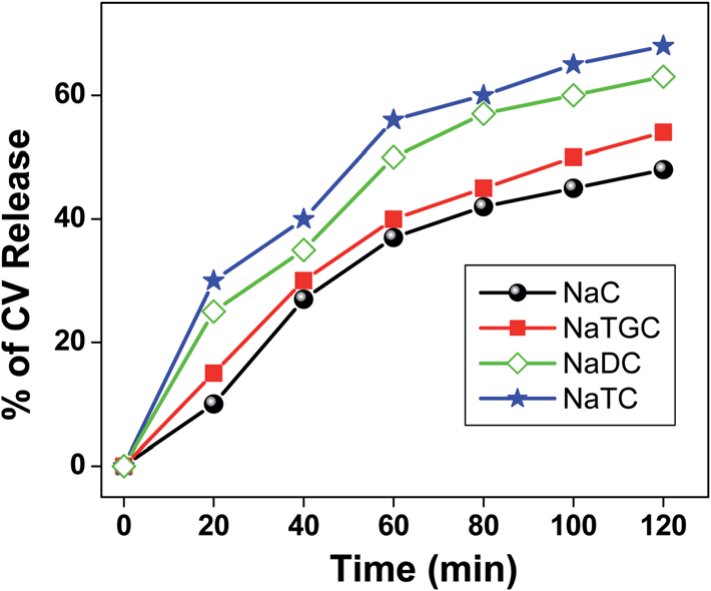
Release kinetics of CV molecule from different bile salts with addition of KCl salt.

Moreover, we have studied the release kinetics of CV molecule encapsulated in different bile salt aggregates with the addition of KCl salts (Fig. 7). It has been found that release of CV molecule follows the order as: NaTC > NaDC > NATGC > NaC.

Aggregation numbers of different bile salt systems were calculated using the following equation:^38^
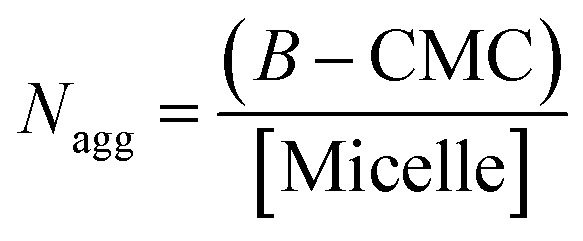
where, ‘*B*’ represents the highest micellar concentration of respective bile-salt at saturation, CMC is the critical micellar concentration.

It has been reported that for conventional surfactants increase in ionic strength, temperature and decrease in pH leads to growth of the micelles. In contrast, bile-salt aggregates do not follow general growth behaviour and their growth depends upon various factors, such as concentration which varies from different bile species.^44–47^ Zana *et al.*^36^ have reported that with the increase in concentration of NaCl salt, there is a prominent increases in the aggregation number due to salting out effect. As a result, the hydrophobic interaction enhances, which results decrease in CMC values and increase the aggregation number. But in our case, a completely opposite trend (Table S1†) was observed. Addition of KCl to the respective CV–bile system leads to decrease in aggregation number. This result clearly confirms that KCl disrupts the bile-aggregates.

## 4. Conclusion

In conclusion, the present work deciphers the photophysics and supramolecular interaction of non-fluorescent drug molecule (CV) in presence of varying the hydrophilic head groups and hydrophobic skeletons of four bile-salts (NaC, NaDC, NaTC and NaTGC). From the UV-vis absorption and fluorescence emission techniques, it is clear that the studied drug molecule (CV) molecule displayed remarkable enhancement in absorption and fluorescence emission spectral behaviour. A significant increase in ‘*Φ*’ value of CV in bile-salt aggregates (∼1000 folds), high ground state and excited state binding constant as well as partition coefficient clearly depicts the dye molecule resides in bile-salt aggregates. The release of the dye molecule from the confined bile-salt aggregates to the aqueous medium was modulated by addition of non-toxic, biodegradable substance (KCl). It has been found that addition of nano-molar concentration of KCl salt to the bile-salt aggregates leads to extreme modification of the photophysical property of CV molecule. The release of the dye molecule was confirmed upon addition of KCl from the decrease in absorption, fluorescence intensity, fluorescence quantum yield, ground state and excited state binding constant values, partition coefficient, percentage of release and aggregation number of CV molecule entrapped in bile-salt aggregates. Therefore, it is perhaps not an overstatement that the work involved in this work might be valuable for potential targeted drug-delivery implications, detection analysis, sensors, and also in physiological systems.

## Conflicts of interest

The authors have no conflict of interest in the publication of this manuscript.

## Supplementary Material

RA-011-D0RA06599D-s001
