# Supplementary material for: Encapsulation and release of non-fluorescent crystal violet confined in bile-salt aggregates
Source: RSC Adv. 2021 Mar 15;11(18):10912–21. doi: 10.1039/d0ra06599d (PMC8695816; doi:10.1039/d0ra06599d)
Supplement: RA-011-D0RA06599D-s001 [file RA-011-D0RA06599D-s001.pdf]

Electronic Supplementary Material (ESI) for RSC Advances.
This journal is © The Royal Society of Chemistry 2021

Supporting Information

Encapsulation and Release of Non-fluorescent Crystal Violet Confined in Bile-salt Aggregates

Prachi Sharma[a], Neeraj Sohal[a], Banibrata Maity*[b]

[a]School of Chemistry and Biochemistry, Thapar Institute of Engineering and Technology, Patiala 147004, India.

[b]School of Chemistry and Biochemistry, Affiliate Faculty-TIET-Virginia Tech Center of Excellence in Emerging Materials, Thapar Institute of Engineering and Technology, Patiala-147004, India

Email: banibrata.maity@thapar.edu, baniiitchem@gmail.com

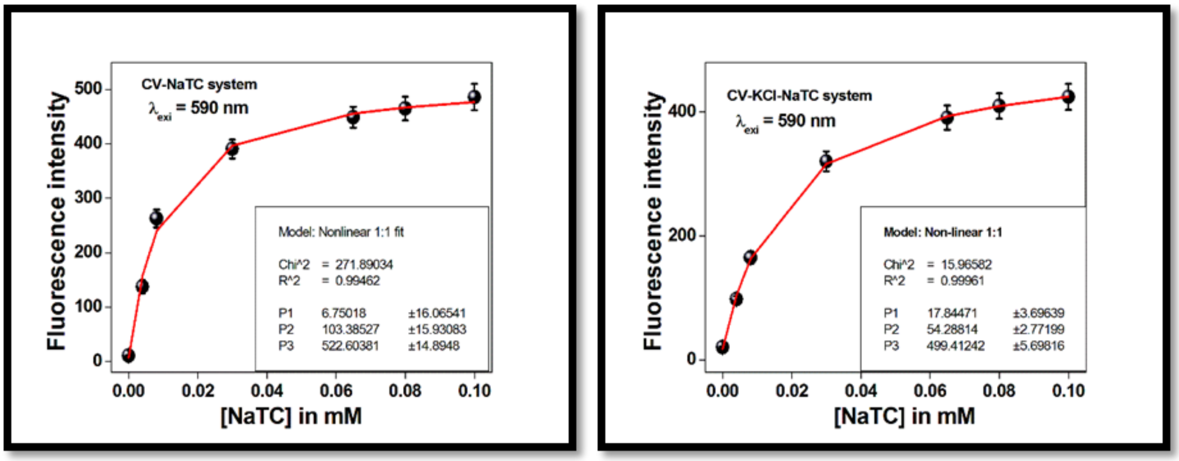

Fig. S1 Excited state binding constant plot of CV-NaTC and CV-KCl-NaTGC at λexi = 590 nm.

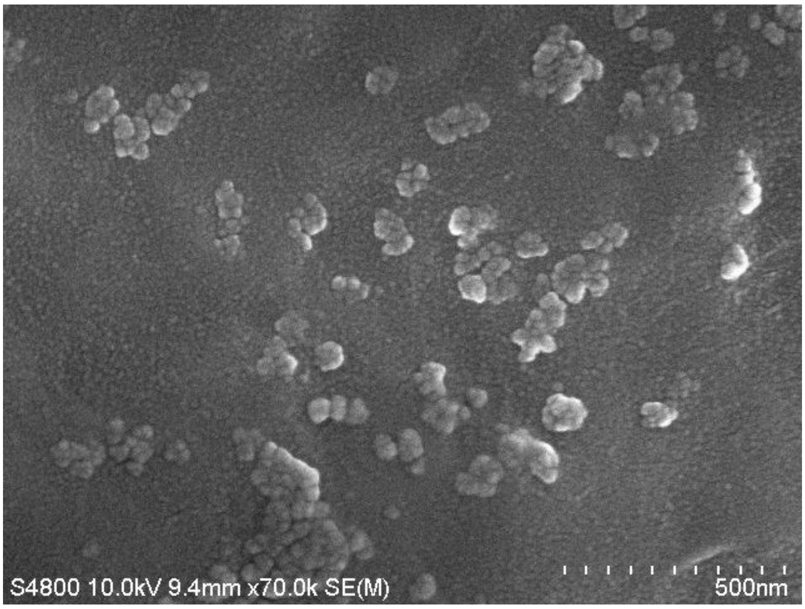

Fig. S2 FESEM image of CV-NaTC complex.

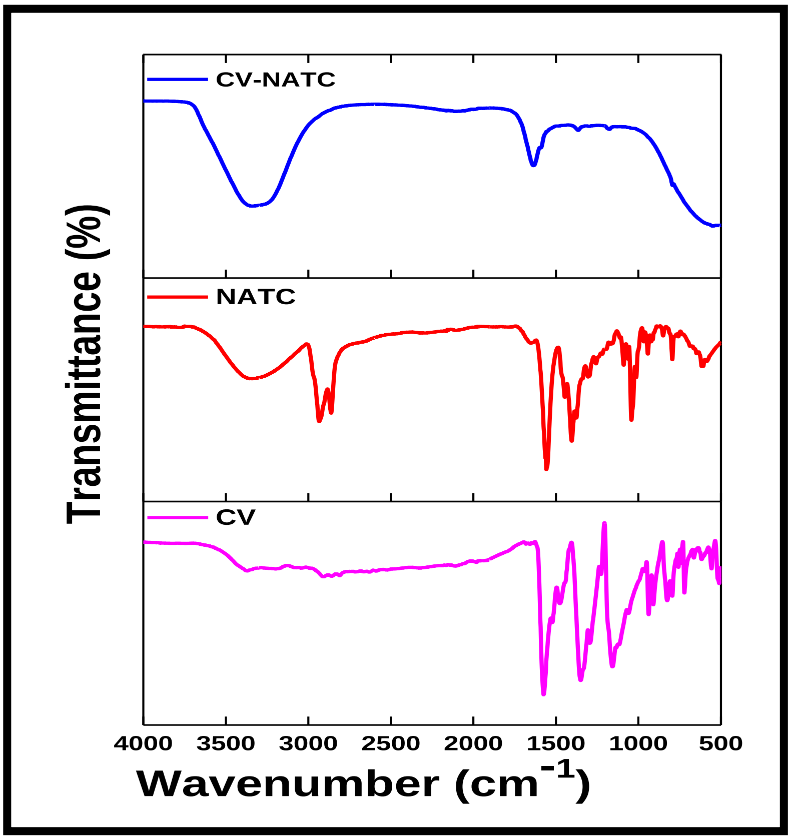

Fig. S3 FTIR spectra of CV, NaTC and CV-NaTC complex.

Table S1 Aggregation number (Nagg) of CV in bile-salt system.

Bile-salt [100mM]	Aggregation number (N_{agg}) in CV-bile - KCl system	Aggregation number (N_{agg}) in CV-KCl - bile system
NaC	4	2
NaDC	4	3
NaTC	3	2
NaTGC	3	2
